# Cholecystokinin Octapeptide Promotes ANP Secretion through Activation of NOX4–PGC-1*α*–PPAR*α*/PPAR*γ* Signaling in Isolated Beating Rat Atria

**DOI:** 10.1155/2022/5905374

**Published:** 2022-06-20

**Authors:** Zhuo-na Han, Xiao-xue Lin, Yue-ying Wang, Ran Ding, Lan Hong, Xun Cui

**Affiliations:** ^1^Department of Physiology, School of Medical Sciences, Yanbian University, Yanji 133-002, China; ^2^Cellular Function Research Center, Yanbian University, Yanji 133-002, China

## Abstract

Atrial natriuretic peptide (ANP), a canonical cardiac hormone, is mainly secreted from atrial myocytes and is involved in the regulation of body fluid, blood pressure homeostasis, and antioxidants. Cholecystokinin (CCK) is also found in cardiomyocytes as a novel cardiac hormone and induces multiple cardiovascular regulations. However, the direct role of CCK on the atrial mechanical dynamics and ANP secretion is unclear. The current study was to investigate the effect of CCK octapeptide (CCK-8) on the regulation of atrial dynamics and ANP secretion. Experiments were performed in isolated perfused beating rat atria. ANP was measured using radioimmunoassay. The levels of hydrogen peroxide (H_2_O_2_) and arachidonic acid (AA) were determined using ELISA Kits. The levels of relative proteins and mRNA were detected by Western blot and RT-qPCR. The results showed that sulfated CCK-8 (CCK-8s) rather than desulfated CCK-8 increased the levels of phosphorylated cytosolic phospholipase A2 and AA release through activation of CCK receptors. This led to the upregulation of NADPH oxidase 4 (NOX4) expression levels and H_2_O_2_ production and played a negative inotropic effect on atrial mechanical dynamics via activation of ATP-sensitive potassium channels and large-conductance calcium-activated potassium channels. In addition, CCK-8s-induced NOX4 subsequently upregulated peroxisome proliferator-activated receptor *γ* (PPAR*γ*) coactivator-1*α* (PGC-1*α*) expression levels through activation of p38 mitogen-activated protein kinase as well as the serine/threonine kinase signaling, ultimately promoting the secretion of ANP via activation of PPAR*α* and PPAR*γ*. In the presence of the ANP receptor inhibitor, the CCK-8-induced increase of AA release, H_2_O_2_ production, and the upregulation of NOX4 and CAT expressions was augmented but the SOD expression induced by CCK-8s was repealed. These findings indicate that CCK-8s promotes the secretion of ANP through activation of NOX4–PGC-1*α*–PPAR*α*/PPAR*γ* signaling, in which ANP is involved in resistance for NOX4 expression and ROS production and regulation of SOD expression.

## 1. Introduction

Atrial natriuretic peptide (ANP), as a cardiac hormone, is synthesized and secreted mainly from atrial myocytes in response to stretch and other stimuli; it is primarily involved in the regulation of body fluid volume and blood pressure [1–3]. In addition, ANP has anti-ischemic [4], anti-inflammatory [5], antihypertrophic [6], and anticancer [7] properties. Although very little is known about the relationship between ANP and reactive oxygen species (ROS) production under physiological conditions or during the development of cardiovascular disease, ANP is associated with important antioxidant defense in cardiomyocytes and vascular cells [5].

Cholecystokinin (CCK) is a classical gut hormone and a potent stimulator of gallbladder contraction that was found in extracts of small intestinal mucosa in the 1920s [8, 9]. Subsequently, CCK has also been found in neurons [10], immune cells [11], kidney cells, and lung cells [12]. Pro-CCK is processed into several molecular forms such as CCK-58, CCK-33, CCK-22, CCK-8, and CCK-4; however, sulfated carboxyl-terminal CCK octapeptide (CCK-8) is the major biological active fragment of CCK, which retains most of the bioactivities of CCK. Two types of receptors for CCK (CCK_1_ receptor (CCK_1_R) and CCK_2_ receptor (CCK_2_R)) have been classified as belonging to the G protein-coupled receptor superfamily (GPCRs) and its distribution is tissue-dependent [12–14]. Recently, it has been demonstrated that pro-CCK, CCK, and its receptors are expressed in mammalian cardiomyocytes [15, 16]. Studies have shown that CCK has physiological roles in regulating blood pressure [17] and heart rate [15] and can enhance cardiac contractility [18]. In addition, CCK can alleviate fibrosis in the noninfarcted regions and delay the left ventricular remodeling and the progress of heart failure in left coronary artery ligation induced myocardial infarction of rats [19]. Nevertheless, the development of postinfarction heart failure is associated with the upregulation of CCK levels in relation to cardiac functional parameters and brain natriuretic peptide levels [20]. Furthermore, mRNA and protein levels of CCK are increased in the hypertrophic myocardial tissue of rats, which was positively correlated with changes in the left ventricular wall thickness [12]. In clinical studies of heart failure patients, an increase in plasma CCK is associated with mortality in elderly females [21]. Thus, CCK signaling induces multiple effects on cardiovascular physiology and pathophysiology. Still, the direct roles of CCK on the regulation of atrial mechanical dynamics and ANP secretion are not yet identified.

Nicotinamide adenine dinucleotide phosphate oxidases (NOXs), a major enzymatic source of ROS in the cardiovascular system, are activated in specific cardiovascular diseases [22]. Previously, we have found that NOX4, controlled by phospholipase A2 (PLA2), is involved in the regulation of ANP secretion in isolated beating rat atria under hypoxic conditions [23]. Moreover, it has been confirmed that the insensitivity of the CCK_1_R inositol phosphate signaling to pertussis toxin (PTX) indicates that it couples through the Gq family of the G proteins, thereby linking with the PLA2–arachidonic acid (AA) pathway to mediate calcium oscillation and amylase secretion [24, 25]. In contrast, CCK_2_R is coupled to two pathways through PTX-sensitive and PTX-insensitive G proteins, resulting in the regulation of AA release by PTX-sensitive G proteins [24, 26] and mitogen-activated protein kinase (MAPK) signaling pathways [24, 27]. Owing to the effect of PLA2–AA signaling on NOX4 activity and its role in the regulation of atrial ANP secretion [23, 28], we hypothesize that CCK regulates ANP secretion through activation of NOX4 via PLA2–AA signaling. This study is to verify the hypothesis using CCK-8 in isolated perfused beating rat atria. This study shows that sulfated CCK-8 (CCK-8s) rather than desulfated CCK-8 promotes ANP secretion through activation of NOX4–peroxisome proliferator-activated receptor *γ* (PPAR*γ*) coactivator-1*α* (PGC-1*α*)–PPAR*α*/PPAR*γ* signaling, in which CCK-8s-induced ANP is involved in the resistance for NOX4 expression and ROS production and regulation of SOD expression.

## 2. Materials and Methods

### 2.1. Preparation of a Perfused Beating Rat Atrium Model *In Vitro*

All animal experiments in this study were reviewed and approved by the Institutional Animal Care and Use Committee of Yanbian University and were in accordance with the National Institutes of Health Laboratory Animal Care Guidelines. Specific pathogen-free (SPF) Sprague-Dawley (SD) rats (250~300 g, 18 weeks, male) were obtained from the Laboratory Animal Center of Yanbian University (laboratory animal use license number: SYXK (Ji) 2020-0009) for preparation for the beating rat atrial perfusion model. The environmental temperature for the rats was about 18°C~26°C, the relative humidity was 40%~70%, the light cycle was 12 h light/dark, and water was available ad libitum alongside a standard diet. Sterile normal saline was used to prepare the pentobarbital sodium solution, and SD rats were intraperitoneally anesthetized at a dose of 90 mg/kg. Immediate thoracotomy was performed, and the left atrium of the rat was harvested and fixed on a self-made beating rat atrial perfusion device. Each rat left atrium was subjected to atrial electrical stimulation, and a peristaltic pump was used to infuse HEPES buffer and the reagents required for the experiment into the atria at a constant rate of 1.0 mL/min. A continuous oxygen supply was maintained along with an atrial temperature of 36°C. HEPES buffer contained (in mM) 118.0 NaCl, 4.7 KCl, 2.5 CaCl_2_, 1.2 MgCl_2_, 25.0 NaHCO_3_, 10.0 glucose, 10.0 HEPES (pH 7.4 with NaOH), and 0.1% BSA.

### 2.2. Experimental Protocols and Treatment Reagents

#### 2.2.1. Experimental Grouping and Treatment Reagents

Rats were randomized into 18 different groups: (1) control; (2) CCK-8s (sulfated cholecystokinin octapeptide, 100.0 pM; AS-200741; Eurogentec, Germany); (3) CCK-8d (desulfated cholecystokinin octapeptide, 100.0 pM; HY-P0196A; MedChemExpress, USA); (4) Loxiglumid (an antagonist of CCK_1_R, 0.5 mM; HY-B2154; MedChemExpress, USA) + CCK-8s (100.0 pM); (5) YM022 (an antagonist of CCK_2_R, 70.0 pM; HY-103355; MedChemExpress, USA) + CCK-8s (100.0 pM); (6) U-73122 (an inhibitor of phospholipase C (PLC), 10.0 *μΜ*; HY-13419; MedChemExpress, USA) + CCK-8s (100.0 pM); (7) CAY10650 (an inhibitor of cytosolic phospholipase A2 (cPLA2), 120.0 nM; HY-10801; MedChemExpress, USA) + CCK-8s (100.0 pM); (8) GLX351322 (an inhibitor of NADPH oxidase 4 (NOX4), 25.0 *μΜ*; HY-100.0111; MedChemExpress, USA) + CCK-8s (100.0 pM); (9) GLX351322 (25.0 *μΜ*); (10) Glibenclamide (an inhibitor of ATP-sensitive potassium (K_ATP_) channel, 0.1 m*Μ*; PHR1287; Sigma-Aldrich, USA) + CCK-8s (100.0 pM); (11) GAL-021 (a blocker of large-conductance calcium-activated potassium (BK_Ca_), 30.0 *μΜ*; HY-101422; MedChemExpress, USA) + CCK-8s (100.0 pM); (12) SB239063 (an antagonist of p38 MAPK, 15.0 *μ*M; HY-11068; MedChemExpress, USA) + CCK-8s (100.0 pM); (13) LY294002 (an inhibitor of serine/threonine kinase (Akt), 10.0 *μΜ*; HY-10108; MedChemExpress, USA) + CCK-8s (100.0 pM); (14) SR-18292 (an antagonist of peroxisome proliferator-activated receptor *γ* (PPAR*γ*) coactivator-1*α* (PGC-1*α*), 50.0 *μΜ*; HY-101491; MedChemExpress, USA) + CCK-8s (100.0 pM); (15) GW6471 (an antagonist of PPAR*α*, 10.0 *μ*M; HY-15372; MedChemExpress, USA) + CCK-8s (100.0 pM); (16) GW9662 (an inhibitor of PPAR*γ*, 0.1 *μ*M; HY-16578; MedChemExpress, USA) + CCK-8s (100.0 pM); (17) A71915 (an antagonist of ANP, 0.3 *μΜ*; SML2908; Sigma-Aldrich, USA) + CCK-8s (100.0 pM); (18) A71915 (0.3 *μΜ*).

#### 2.2.2. Experimental Protocols

Each left atrium was first stably perfused for 60 min, followed by six cycles of reperfusion according to the experimental group, each cycle for 12 min, as shown in Table 1. To evaluate the effects of CCK-8 on atrial pulse pressure and ANP secretion, two cycles of control period were followed by infusion of CCK-8s or CCK-8d for four cycles. In another series of experiments, one cycle of the control period was followed by one cycle of treatment reagents and then CCK-8s for four cycles in the continuous presence of the treatment reagents. To test the effects of GLX and A71 on the atrial pulse pressure and ANP secretion, the control period was followed by five cycles of GLX or A71. All atria were harvested immediately after the end of perfusion and cryopreserved at -80°C for subsequent experiments.

### 2.3. Determination of ANP and Pulse Pressure

As previously described [23], ANP levels of perfusates were detected using an Iodine [^125^I] Atrial Natriuretic Factor Radioimmunoassay Kit (North Institute of Biological Technology, Beijing, China). Changes in atrial pulse pressure were recorded using a multichannel physiological signal acquisition system (RM6240BD, 1.5 Hz, 0.3 ms, 35.0 V; Chengdu, China) via a baroreceptor (Statham P23Db; Oxnard, CA, USA).

### 2.4. ELISA

The levels of hydrogen peroxide (H_2_O_2_) and AA in equal volumes of rat left atrial lysis solutions were determined using the Rat H_2_O_2_ ELISA Kit and Rat AA ELISA Kit; these kits were purchased from SinoBestBio (Shanghai, China)

### 2.5. Western Blot Analysis and Antibodies

Left atria were lysed and homogenized adequately using RIPA buffer (high) (R0010, Solarbio Science & Technology; Beijing, China) in an ice bath. Protein quantification was then performed using an Enhanced BCA Protein Assay Kit (P0009, Beyotime; Shanghai, China). The samples were subjected to protein denaturation with 5× sodium dodecyl sulfate-polyacrylamide gel electrophoresis (SDS-PAGE) loading buffer (ComWin Biotech; Beijing, China). Denatured samples were electrophoresed in 8% or 15% SDS-PAGE gels at room temperature. After electrophoresis, the separated proteins were transferred from the gel to a polyvinylidene fluoride (PVDF) membrane using the wet transfer method and transferred for 60 min or 90 min at 4°C. The blotted membranes were blocked with 5% nonfat dry milk in PBS for 2 h at room temperature, followed by incubation with primary antibodies overnight at 4°C. The next day, after incubation with secondary antibodies for 2 h, the membranes were washed for 30 min and developed using a sensitive ECL chemiluminescence detection kit (PK10002, Proteintech; Wuhan, China). The bands were analyzed grayscale using ImageJ (National Institutes of Health; Bethesda, USA) software, and the results of the analysis were normalized.

The following antibodies were used in this experiment: an antibody of CCK_1_R (DF4914), an antibody of CCK_2_R (DF2793), an antibody of phospho-cPLA2 (Ser505) (AF3329), an antibody of cPLA2 (AF6329), an antibody of NOX4 (DF6924), an antibody of PGC-1*α* (AF5395), an antibody of phospho-PPAR*α* (Ser12) (AF8392), an antibody of PPAR*α* (AF5301), an antibody of phospho-PPAR*γ* (Ser112) (AF3284), an antibody of PPAR*γ* (AF6284), an antibody of superoxide dismutase (SOD) (AF5144), an antibody of catalase (CAT) (DF7545), an antibody of glutathione peroxidase (GPX) (DF6701), an antibody of phospho-pan-AKT1/2/3 (Ser473) (AF0016), and an antibody of pan-AKT1/2/3 (AF6261) were all purchased from Affinity Biosciences (Jiangsu, China). An antibody of phospho-p38 MAPK (Thr180/Tyr182) polyclonal (28796-1-AP), an antibody of p38 MAPK polyclonal (14064-1-AP), and HRP goat anti-rabbit IgG were all bought from Proteintech (Wuhan, China). Natriuretic peptide A (NPPA) rabbit pAb (A14755) and *β*-actin rabbit mAb (High Dilution) (AC026) were from ABclonal Technology (Wuhan, China).

### 2.6. RT-qPCR

In brief, total RNA was extracted from left atrial tissues using an RNApure Total RNA Kit (RN03, Aidlab; Beijing, China). The first strand of cDNA was then synthesized by high-performance reverse transcription using a SweScript RT I First Strand cDNA Synthesis Kit (G3330, Servicebio; Wuhan, China). Finally, the real-time quantitative polymerase chain reaction (RT-qPCR) was performed with 2× SYBR Green qPCR Master Mix (High ROX) (G3322, Servicebio; Wuhan, China). Results were calculated using the 2^-*ΔΔ*CT^ relative quantification method and were normalized. Primer sequences are shown in Table 2.

### 2.7. Statistical Analysis

Statistical analysis was performed by one-way ANOVA and two-way ANOVA with Tukey's multiple comparison test using GraphPad Prism 9.0 (GraphPad Software; San Diego, USA). All data were normally distributed (Kolmogorov-Smirnov test) and given as mean ± standard error of the mean (SEM). *P* < 0.05 was considered statistically significant.

## 3. Results

### 3.1. Effects of CCK-8 on the ANP Secretion and Atrial Dynamics

**Figure 1:**
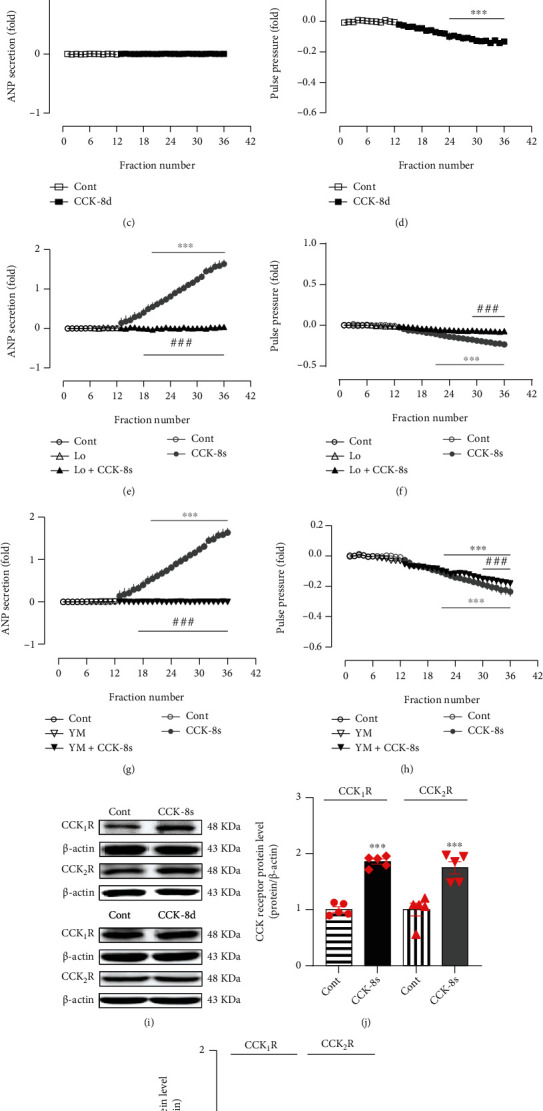
CCK-8 role in ANP secretion and dynamics and its effects on CCK receptor expressions in isolated beating rat atria. (a, c, e, g) Atrial ANP secretion by radioimmunoassay. (b, d, f, h) Atrial pulse pressure by RM6240BD. (i) The protein expression levels of CCK_1_R and CCK_2_R were detected by Western blot. (j, k) The statistics histograms of Western blot were expressed as band density normalized versus *β*-actin. Cont: control; CCK-8s: sulfated CCK-8; CCK-8d: desulfated CCK-8; Lo: Loxiglumid, an antagonist of CCK_1_ receptor; YM: YM022, an antagonist of CCK_2_ receptor. Data were expressed as mean ± SEM. (a–h) *n* = 6; (i–k) *n* = 5. ^∗∗∗^*P* < 0.001 vs. control period or group; ^###^*P* < 0.001 vs. CCK-8s period or group; ns: no significant.

To determine the effects of CCK-8 on atrial dynamics and ANP secretion, CCK-8s and CCK-8d were used in the experiments. In isolated beating rat atria, the infusion of CCK-8s significantly increased ANP secretion and inhibition of atrial pulse pressure (*P* < 0.001 vs. control period; Figures 1(a) and 1(b)) in a time-dependent manner. Meanwhile, CCK-8d obviously inhibited the atrial pulse pressure (*P* < 0.001 vs. control period; Figure 1(d)) but did not affect the ANP secretion (Figure 1(c)). In the presence of CCK_1_R and CCK_2_R antagonists, Loxiglumid and YM022, the CCK-8s-induced increase of ANP secretion was abolished completely (*P* < 0.001 vs. CCK-8s alone period; Figures 1(e) and 1(g)). The CCK-8s-induced inhibition of atrial pulse pressure was almost completely blocked by Loxiglumid (*P* < 0.001 vs. CCK-8s alone period; Figure 1(f)) and notably attenuated by YM022 (*P* < 0.001 vs. CCK-8s alone period; Figure 1(h)). In addition, the expressions of CCK_1_R and CCK_2_R were significantly upregulated in atrial tissue after infusion of CCK-8s rather than CCK-8d (*P* < 0.001 vs. control group; Figures 1(i)–1(k)). The results indicated that sulfated rather than desulfated CCK-8 was able to promote ANP secretion through CCK receptors and has a negative inotropic effect on the mechanical dynamics of isolated perfused beating rat atria.

### 3.2. Effects of PLC and cPLA2 on CCK-8s-Induced ANP Secretion

**Figure 2:**
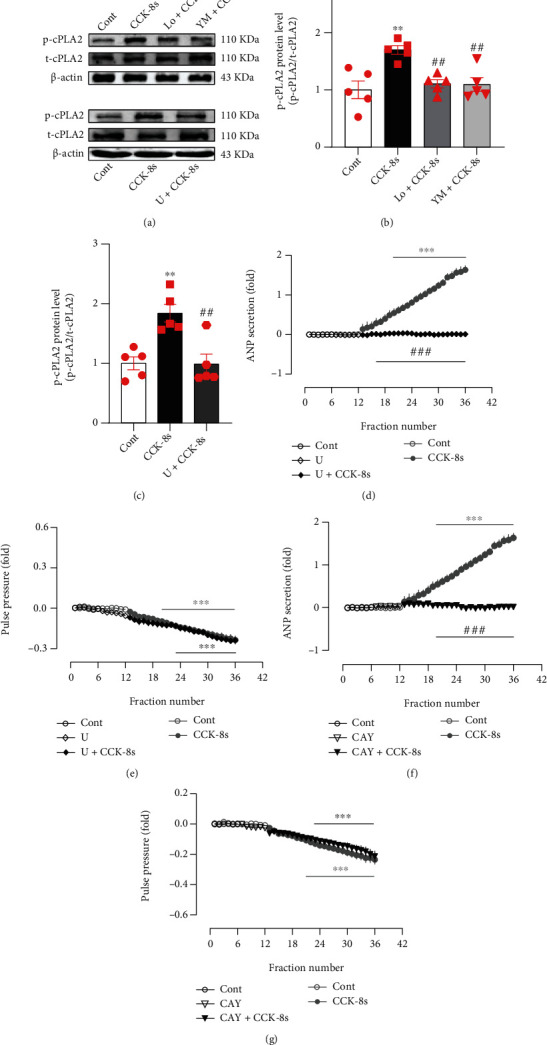
Effects of CCK-8s and PLC on cPLA2 expression and its role in ANP secretion and dynamics in isolated beating rat atria. (a) The protein expression levels of p-cPLA2 were detected by Western blot. (b, c) The statistics histograms of Western blot were expressed as band density normalized versus t-cPLA2. (d, f) Atrial ANP secretion by radioimmunoassay. (e, g) Atrial pulse pressure by RM6240BD. Cont: control; CCK-8s: sulfated CCK-8; Lo: Loxiglumid, an antagonist of CCK_1_ receptor; YM: YM022, an antagonist of CCK_2_ receptor; U: U-73122, an inhibitor of PLC; CAY: CAY10650, an inhibitor of cPLA2. Data were expressed as mean ± SEM. (a–c) *n* = 5; (d–g) *n* = 6. ^∗∗^*P* < 0.01 and ^∗∗∗^*P* < 0.001 vs. control period or group; ^##^*P* < 0.01 and ^###^*P* < 0.001 vs. CCK-8s period or group.

CCK receptors couple Gq proteins to PLC and PLA2, subsequently leading to downstream signaling [24–27]. Therefore, to investigate the mechanism by which CCK-8s promotes ANP secretion, the effects of CCK-8s on the levels of p-cPLA2 and its role in ANP secretion were observed. As shown in Figure 2, the levels of p-cPLA2 were markedly increased in atrial tissue after infusion of CCK-8s (*P* < 0.01 vs. control group; Figures 2(a)–2(c)), which were blocked by pretreatment with antagonists of CCK receptors (*P* < 0.01 vs. CCK-8s group; Figures 2(a) and 2(b)) and U73122, an inhibitor of PLC (*P* < 0.01 vs. CCK-8s group; Figures 2(a) and 2(c)). In addition, infusion of the CCK-8s increased ANP secretion which was also prevented by pretreatment with U73122 (*P* < 0.001 vs. CCK-8s alone period; Figure 2(d)) and CAY10650, an inhibitor of cPLA2 (*P* < 0.001 vs. CCK-8s alone period; Figure 2(f)), while CCK-8s-induced inhibition of pulse pressure was not affected by U73122 and CAY10650 (Figures 2(e) and 2(g)). The data suggested that PLC and cPLA2 mediate the process of CCK-8s-induced increase of atrial ANP secretion.

### 3.3. Effects of CCK-8s on NOX4 Expression and Its Role in ANP Secretion

**Figure 3:**
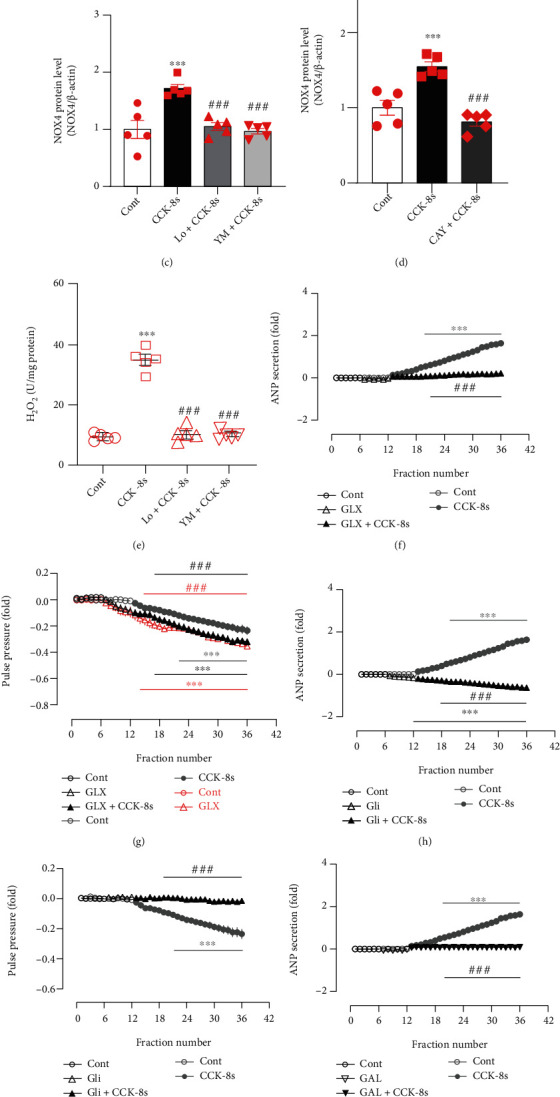
Effects of CCK-8s on NOX4 expression and its role in ANP secretion and dynamics in isolated beating rat atria. (a) The level of AA was tested by the ELISA method. (b) The protein expression levels of NOX4 were detected by Western blot. (c, d) The statistics histograms of Western blot were expressed as band density normalized versus *β*-actin. (e) The level of H_2_O_2_ was tested by ELISA. (f, h, j) Atrial ANP secretion by radioimmunoassay. (g, i, k) Atrial pulse pressure by RM6240BD. Cont: control; CCK-8s: sulfated CCK-8; Lo: Loxiglumid, an antagonist of CCK_1_ receptor; YM: YM022, an antagonist of CCK_2_ receptor; U: U-73122, an inhibitor of PLC; CAY: CAY10650, an inhibitor of cPLA2; GLX: GLX351322, an inhibitor of NOX4; Gli: Glibenclamide, an inhibitor of K_ATP_; GAL: GAL-021, a blocker of BK_Ca_. Data were expressed as mean ± SEM. (a–e) *n* = 5; (f–k) *n* = 6. ^∗∗∗^*P* < 0.001 vs. control period or group; ^##^*P* < 0.01 and ^###^*P* < 0.001 vs. CCK-8s period or group.

According to the role of CCK-8s on the levels of p-cPLA2 and the effect of cPLA2 on AA release and NOX4 activation [29], the levels of AA, NOX4 expression, and H_2_O_2_ production induced by CCK-8s were observed. Results revealed that the levels of AA were remarkably increased in atrial tissue after infusion of CCK-8s (*P* < 0.001 vs. control group; Figure 3(a)), which were blocked by pretreatment with antagonists of CCK receptors and U73122 (*P* < 0.001 and *P* < 0.01 vs. CCK-8s group; Figure 3(a)). In addition, the expression of NOX4 was dramatically upregulated in CCK-8s-infused atrial tissue (*P* < 0.001 vs. control group; Figures 3(b)–3(d)); this effect was blocked by antagonists of CCK receptors and CAY10650 pretreatment (*P* < 0.001 vs. CCK-8s group; Figures 3(b)–3(d)). The levels of H_2_O_2_ were also markedly increased in atrial tissue after infusion of CCK-8s (*P* < 0.001 vs. control group; Figure 3(e)), which were abolished by pretreatment with antagonists of CCK receptors (*P* < 0.001 vs. CCK-8s group; Figure 3(e)). Furthermore, in the presence of GLX351322, an inhibitor of NOX4, CCK-8s-induced increase of ANP secretion was not observed (*P* < 0.001 vs. CCK-8s alone period; Figure 3(f)), and the inhibition of atrial pulse pressure was also removed (*P* < 0.001 vs. control period and CCK-8s alone period; *P* > 0.05 vs. GLX351322 alone period; Figure 3(g)). In the presence of Glibenclamide, an inhibitor of K_ATP_, the effects of CCK-8s on atrial ANP secretion and mechanical dynamics were removed (*P* < 0.001 vs. CCK-8s alone period, respectively; Figures 3(h) and 3(i)), and a blocker of BK_Ca_, GAL-021, mimicked the role of Glibenclamide (*P* < 0.001 vs. CCK-8s alone period, respectively; Figures 3(j) and 3(k)). These results suggested that CCK-8s upregulated the expression of NOX4 via cPLA2–AA signaling, thereby increasing the secretion of ANP and exerting a negative inotropic effect on atrial mechanical dynamics, in which K_ATP_ and BK_Ca_ were involved.

### 3.4. Effects of CCK-8s-Induced NOX4 on p38 MAPK and Akt Expressions

**Figure 4:**
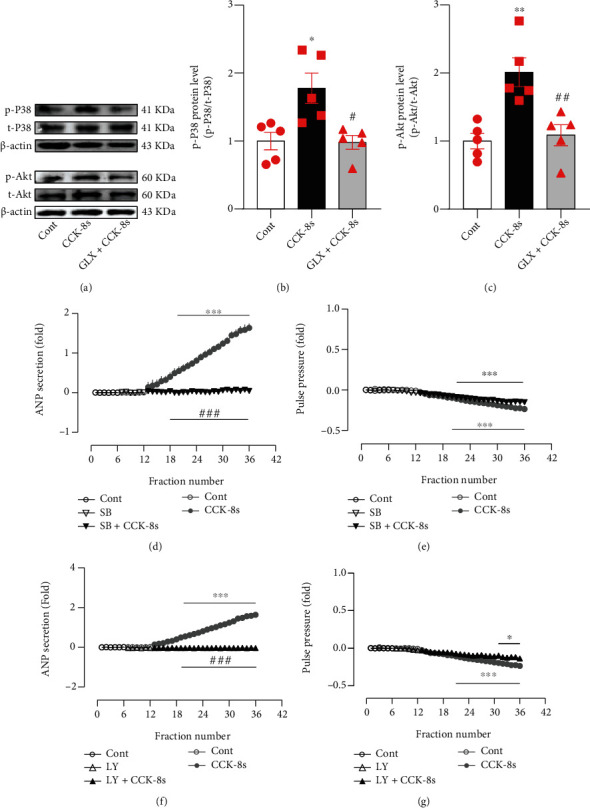
Effects of p38 MAPK and Akt on ANP secretion and dynamics induced by CCK-8s in isolated beating rat atria. (a) The protein expression levels of p-p38 MAPK and p-Akt were detected by Western blot. (b) The statistics histograms of Western blot were expressed as band density normalized versus t-p38. (c) The statistics histograms of Western blot were expressed as band density normalized versus t-Akt. (d, f) Atrial ANP secretion by radioimmunoassay. (e, g) Atrial pulse pressure by RM6240BD. Cont: control; CCK-8s: sulfated CCK-8; GLX: GLX351322, an inhibitor of NOX4; SB: SB239063, an antagonist of p38 MAPK; LY: LY294002, an inhibitor of Akt. Data were expressed as mean ± SEM. (a–c) *n* = 5; (d–g) *n* = 6. ^∗^*P* < 0.05, ^∗∗^*P* < 0.01, and ^∗∗∗^*P* < 0.001 vs. control period or group; ^#^*P* < 0.05, ^##^*P* < 0.01, and ^###^*P* < 0.001 vs. CCK-8s period or group.

In accordance with the relationship between the activation of the NOX4-dependent p38 MAPK axis and cell oxidative damage [30], the effects of CCK-8s-induced NOX4 on the p38 MAPK and Akt protein expression in atrial tissue after infusion of CCK-8s were observed. The data showed that CCK-8s notably increased the levels of phosphorylated p38 (p-p38) MAPK, (*P* < 0.05 vs. control group; Figures 4(a) and 4(b)) and phosphorylated Akt (p-Akt) (*P* < 0.01 vs. control group; Figures 4(a) and 4(c)), which were eliminated by GLX351322 (*P* < 0.05 and *P* < 0.01 vs. CCK-8s group; Figures 4(a)–4(c)). Moreover, the CCK-8s-induced ANP secretion was blocked by inhibitors of p38 MAPK and Akt, SB239063, and LY294002 (*P* < 0.001 vs. CCK-8s alone period; Figures 4(d) and 4(f)), while the inhibition of atrial pulse pressure induced by CCK-8s was not notably changed by SB239063 and LY294002 (Figures 4(e) and 4(g)). These results demonstrated that p38 MAPK and Akt controlled by NOX4 were involved in the regulation of CCK-8s-induced ANP secretion.

### 3.5. Effects of CCK-8s on the Expressions of PGC-1*α* and PPAR*α* as well as PPAR*γ*

**Figure 5:**
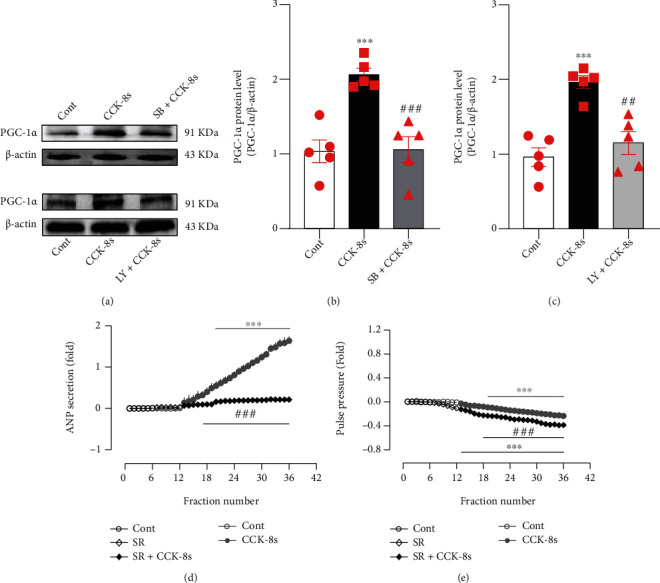
Effects of CCK-8s on PGC-1*α* expression and its role on ANP secretion and dynamics in isolated beating rat atria. (a) The protein expression levels of PGC-1*α* were detected by Western blot. (b, c) The statistics histograms of Western blot were expressed as band density normalized versus *β*-actin. (d) Atrial ANP secretion by radioimmunoassay. (e) Atrial pulse pressure by RM6240BD. Cont: control; CCK-8s: sulfated CCK-8; SB: SB239063, an antagonist of p38 MAPK; LY: LY294002, an inhibitor of Akt; SR: SR-18292, an antagonist of PGC-1*α*. Data were expressed as mean ± SEM. (a–c) *n* = 5; (d, e) *n* = 6. ^∗∗∗^*P* < 0.001 vs. control period or group; ^##^*P* < 0.01 and ^###^*P* < 0.001 vs. CCK-8s period or group.

To investigate the downstream mechanism that p38 MAPK and Akt regulate the CCK-8s-induced increase of ANP secretion, effects of CCK-8s on the expressions of PGC-1*α* and PPAR*α* as well as PPAR*γ* were determined. CCK-8s notably upregulated the expression of PGC-1*α* (*P* < 0.001 vs. control group; Figures 5(a)–5(c)), which was abolished by SB239063 and LY294002 (*P* < 0.001 and *P* < 0.01 vs. CCK-8s group respectively; Figures 5(a)–5(c)). In the presence of SR18292, an antagonist of PGC-1*α*, the CCK-8s-induced increase of ANP secretion was not observed (*P* < 0.001 vs. CCK-8s alone period; Figure 5(d)), while the inhibition of pulse pressure induced by CCK-8s was augmented (*P* < 0.001 vs. CCK-8s alone period; Figure 5(e)).

**Figure 6:**
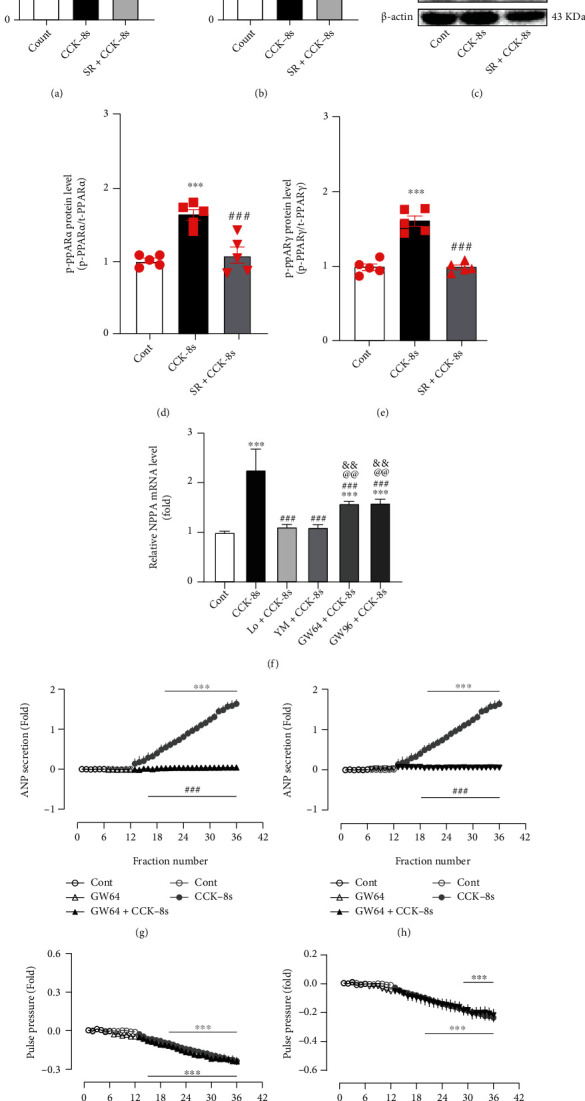
Effects of CCK-8s on PPAR*α* as well as PPAR*γ* expression and its role in ANP secretion and dynamics in isolated beating rat atria. (a, b, f) The mRNA levels of PPAR*α*, PPAR*γ*, and NPPA were tested by RT-qPCR. (c) The protein expression levels of p-PPAR*α* and p-PPAR*γ* were detected by Western blot. (d) The statistics histograms of Western blot were expressed as band density normalized versus t-PPAR*α*. (e) The statistics histograms of Western blot were expressed as band density normalized versus t-PPAR*γ*. (g, h) Atrial ANP secretion by radioimmunoassay. (i, j) Atrial pulse pressure by RM6240BD. Cont: control; CCK-8s: sulfated CCK-8; SR: SR-18292, an antagonist of PGC-1*α*; Lo: Loxiglumid, an antagonist of CCK_1_ receptor; YM: YM022, an antagonist of CCK_2_ receptor; GW64: GW6471, an antagonist of PPAR*α*; GW96: GW9662, an inhibitor of PPAR*γ*. Data were expressed as mean ± SEM. (a–f) *n* = 5; (g–j) *n* = 6. ^∗^*P* < 0.05, ^∗∗^*P* < 0.01, and ^∗∗∗^*P* < 0.001 vs. control period or group; ^###^*P* < 0.001 vs. CCK-8s period or group; ^@@^*P* < 0.01 vs. Lo+CCK-8s group; ^&&^*P* < 0.01 vs. YM+CCK-8s group.

In addition, CCK-8s noticeably increased the mRNA levels of PPAR*α* and PPAR*γ* (*P* < 0.001 vs. control group; Figures 6(a) and 6(b)), concomitant with upregulation of p-PPAR*α* and p-PPAR*γ* protein expression levels (*P* < 0.001 vs. control group; Figures 6(c)–6(e)). The CCK-8s-induced increases of PPAR*α* and PPAR*γ* mRNA were notably inhibited by SR18292 (*P* < 0.001 vs. CCK-8s group; Figures 6(a) and 6(b)), and the protein expression levels of PPAR*α* and PPAR*γ* were also abolished by SR18292 (*P* < 0.001 vs. CCK-8s group; Figures 6(c)–6(e)). CCK-8s also enhanced the NPPA mRNA levels (*P* < 0.001 vs. control group; Figure 6(f)), which were abrogated by inhibitors of CCK receptors (*P* < 0.001 vs. CCK-8s group; Figure 6(f)). Moreover, inhibitors of PPAR*α* and PPAR*γ*, GW6471 and GW9662, not only markedly inhibited the effect of CCK-8s-enhanced mRNA levels of the NPPA (*P* < 0.001 vs. control group; *P* < 0.001 vs. CCK-8s group; Figure 6(f)), but also abolished the role of CCK-8s on ANP secretion (*P* < 0.001 vs. CCK-8s alone period; Figures 6(g) and 6(h)). However, the inhibition of atrial pulse pressure induced by CCK-8s was not affected by GW6471 and GW9662 (Figures 6(i) and 6(j)). These results demonstrated that PGC-1*α* regulated PPAR*α* and PPAR*γ* were involved in the regulation of CCK-8s-induced increase of ANP secretion.

### 3.6. Effects of Endogenous ANP on NOX4, SOD, and CAT Expressions under CCK-8s Action

**Figure 7:**
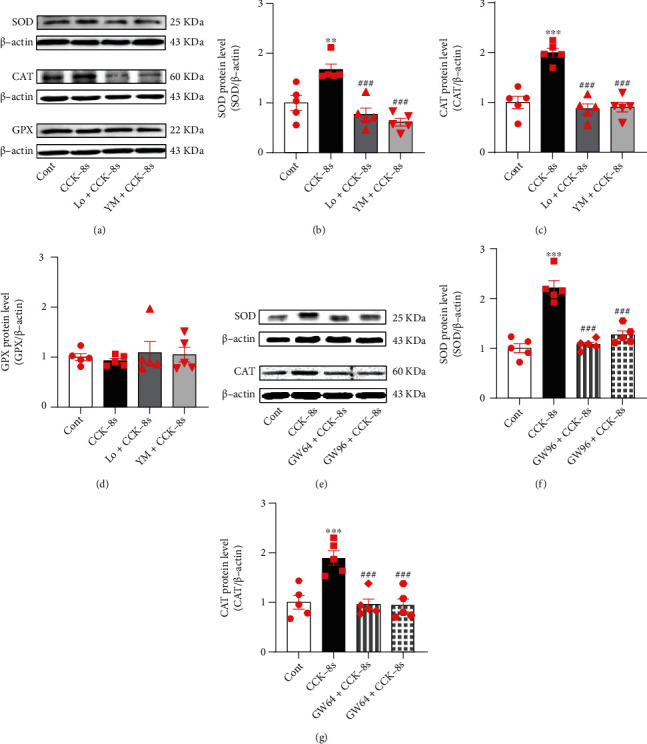
Effects of PPAR*α* and PPAR*γ* on CCK-8s-induced SOD and CAT expressions in isolated beating rat atria. (a) The protein expression levels of SOD, CAT, and GPX were detected by Western blot. (b–d) The statistics histograms of Western blot were expressed as band density normalized versus *β*-actin. (e–g) The bands of SOD and CAT and their statistics histograms. Cont: control; CCK-8s: sulfated CCK-8; Lo: Loxiglumid, an antagonist of CCK_1_ receptor; YM: YM022, an antagonist of CCK_2_ receptor; GW64: GW6471, an antagonist of PPAR*α*; GW96: GW9662, an inhibitor of PPAR*γ*. Data were expressed as mean ± SEM. (a–g) *n* = 5. ^∗∗^*P* < 0.01 and ^∗∗∗^*P* < 0.001 vs. control group; ^###^*P* < 0.001 vs. CCK-8s group.

**Figure 8:**
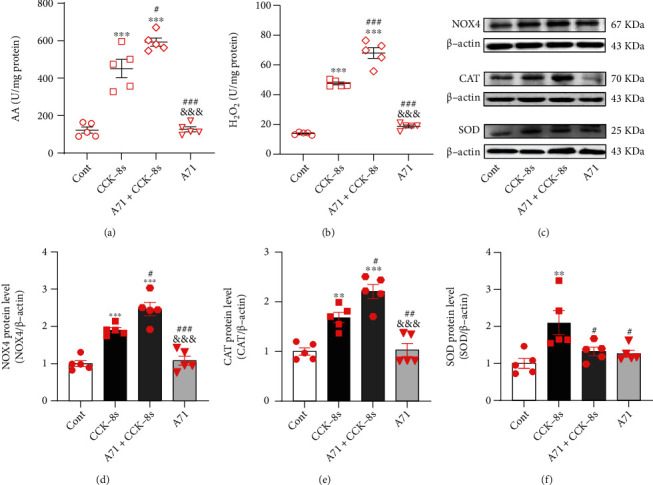
Effects of endogenous ANP on SOD, CAT, and NOX4 expressions under CCK-8s action in isolated beating rat atria. (a, b) The levels of AA and H_2_O_2_ were tested by ELISA method. (c) The protein expression levels of NOX4, CAT, and SOD were detected by Western blot. (d–f) The statistics histograms of Western blot were expressed as band density normalized versus *β*-actin. Cont: control; CCK-8s: sulfated CCK-8; A71: A71915, an antagonist of ANP receptor. Data were expressed as mean ± SEM. (a–f) *n* = 5. ^∗∗^*P* < 0.01 and ^∗∗∗^*P* < 0.001 vs. control group; ^#^*P* < 0.05, ^##^*P* < 0.01, and ^###^*P* < 0.001 vs. CCK-8s group; ^&&&^*P* < 0.001 vs. A71+CCK-8s group.

To define the effects of endogens ANP on NOX4, SOD, and CAT protein expressions under CCK-8s action, another series of experiments were performed with an ANP receptor antagonist. Results showed that CCK-8s significantly increased SOD and CAT rather than GPX protein expression levels (*P* < 0.01 and *P* < 0.001 vs. control group; Figures 7(a)–7(d)); these effects were blocked by inhibitors of CCK receptors and PPAR*α* as well as PPAR*γ* (*P* < 0.001 vs. CCK-8s group; Figures 7(a)–7(c) and 7(e)–7(g)). In the presence of an antagonist of the ANP receptor, A71915, the CCK-8s-induced increase of AA release and H_2_O_2_ levels were further augmented (*P* < 0.05 and *P* < 0.001 vs. CCK-8s group; Figures 8(a) and 8(b)). In addition, A71915 further enhanced the effects of CCK-8s-induced increase of NOX4 and CAT protein expression levels (*P* < 0.05 vs. CCK-8s group; Figures 8(c)–8(e)), but the CCK-8s-induced increase of SOD protein expression was abolished by A71915 (*P* < 0.05 vs. CCK-8s group; Figures 8(c) and 8(f)). Data showed that CCK-8s upregulated the expression of SOD and CAT through activation of PPAR*α* as well as PPAR*γ*. Moreover, endogenous ANP was involved in the inhibition of NOX4 expression and H_2_O_2_ production and the regulation of SOD activity under the action of CCK-8s.

## 4. Discussion

NOX4 is one of the enzymatic sources of ROS generation in the cardiovascular system expressed in the heart [22]. In addition, the role of PLA2 and AA on the activation of NOXs has been demonstrated [28]. In our previous study, we have demonstrated that activated cPLA2 induced by endothelin-1 was involved in the regulation of hypoxia-induced ANP secretion through activation of NOX4 and H_2_O_2_ production in isolated beating rats' atria [23]. The increased NOX4 activity in response to a high pacing frequency also promoted ANP secretion in rat atria [31]. These results suggest that NOX4 is one of the regulatory factors for the secretion of ANP in the atria.

In the current study, CCK-8s rather than CCK-8d significantly upregulated the expression of CCK_1_ and CCK_2_ receptors and increased the levels of p-cPLA2, AA release, NOX4 relative protein levels, and H_2_O_2_ production, concomitant with an increase of ANP secretion and inhibition of mechanical dynamics in isolated beating rat atria. The CCK-8s-induced increases of p-cPLA2 expression and AA release were blocked by inhibitors of CCK receptors and PLC, respectively. Similarly, the CCK-8s-induced upregulation of NOX4 expression and H_2_O_2_ production was abolished by antagonists of CCK receptors, and an inhibitor of cPLA2 also nullified the effect of CCK-8s on NOX4 expression. Furthermore, CCK-8s-induced promotion of ANP secretion was blocked by antagonists of CCK receptors, PLC and cPLA2, respectively, without changes in the inhibition of atrial mechanical dynamics induced by CCK-8s. Nevertheless, CCK-8s-induced inhibition of atrial dynamics was abolished by inhibitors of NOX4, K_ATP_, and BK_Ca_, which was accompanied by blockage of CCK-8s-induced ANP secretion. These results demonstrated that CCK-8s (but not CCK-8d) triggered NOX4 activation and increased H_2_O_2_ production through CCK receptor-mediated activation of cPLA2, leading to an increase in ANP secretion and a negative inotropic action, in which K_ATP_ and BK_Ca_ were involved. The results of the current study are similar to those of the previous studies mentioned above and support previous reports that K_ATP_ and BK_Ca_ are regulated by H_2_O_2_[32–34].

The PGC-1*α* is a member of the PGC-1 family that regulates adaptive thermogenesis and mitochondrial function [35]. PGC-1*α* can be directly activated by p38 MAPK [36] and participates in the elimination of ROS in the heart [37, 38]. PPAR*α* and PPAR*γ*, as downstream signal molecules of PGC-1*α*, inhibit NOX and ROS generation by enhancing the activity of SOD as well as CAT, thereby resisting oxidative stress damage [39, 40]. In addition, the promoter region of the human ANP gene located on the short arm of chromosome 1 contains binding sites for many transcription factors, including PPAR*α* and PPAR*γ* [41]. Furthermore, it has been demonstrated that PPAR*γ* is involved in the regulation of ANP secretion in beating rat atria under normoxic or hypoxic conditions [42–44]. These results suggest that the changes in PPAR*α* and PPAR*γ* activity are closely related to the secretion of ANP.

In the current study, CCK-8s obviously increased the levels of p-p38 MAPK and p-Akt simultaneously with the upregulation of PGC-1*α* expression. The CCK-8s-induced increase of p-p38 MAPK and p-Akt levels was blocked by an inhibitor of NOX4, and the CCK-8s-induced upregulation of PGC-1*α* expression was repealed by inhibitors of p38 MAPK and Akt, respectively. In addition, CCK-8s also markedly increased PPAR*α* as well as PPAR*γ* mRNA levels and their phosphorylated protein expressions, concomitant with the promotion of ANP secretion. An inhibitor of PGC-1*α* abolished the CCK-8s-induced increase of phosphorylated PPAR*α* as well as phosphorylated PPAR*γ* levels by attenuation of PPAR*α* as well as PPAR*γ* mRNA levels; it also abrogated the CCK-8s-induced promotion of ANP secretion. Similarly, inhibitors of PPAR*α* as well as PPAR*γ* attenuated the CCK-8s-induced upregulation of NPPA mRNA levels and repealed the promotion of ANP secretion induced by CCK-8s. The current study data indicate that CCK-8s-induced NOX4 upregulates PGC-1*α* expression through phosphorylation of p38 MAPK and Akt, leading to the activation of PPAR*α* as well as PPAR*γ*, thereby promoting the secretion of ANP. The data is similar to those of the previous studies mentioned above.

SOD, CAT, and GPX are important antioxidant defenses that protect biological systems from ROS toxicity. The superoxide anion produced by NOXs can be converted to H_2_O_2_ and O_2_ by the action of SOD, and the H_2_O_2_ is ultimately detoxified into the water by the action of several enzymes, including CAT and GPX [45]. In the current study, CCK-8s significantly increased NOX4 expression and H_2_O_2_ production concomitant with the upregulation of SOD and CAT rather than GPX expression; this was abolished by inhibitors of CCK receptors. The CCK-8s-induced SOD and CAT expressions were also abolished by antagonists of PPAR*α* and PPAR*γ*. Based on the effect of CCK-8s on PPAR*α* and PPAR*γ* activities, it is suggested that the CCK-8s-induced upregulation of SOD and CAT expressions was related to the PPAR activation. In addition, in the presence of an inhibitor of the ANP receptor, the CCK-8-induced increase of AA release, H_2_O_2_ production, and the upregulation of NOX4 as well as CAT expression was dramatically augmented. These results hint that CCK-8s-induced ANP is involved, at least in part, in the inhibition of AA release, NOX4 expression, and H_2_O_2_ production. As for the augmentation of CAT expression induced by CCK-8s under the presence of an inhibitor of the ANP receptor, we considered that is related to further increase of H_2_O_2_ production, as the CAT can detoxify H_2_O_2_ into water depending on the concentration of H_2_O_2_ [45]. Moreover, the inhibitor of the ANP receptor repealed the CCK-8s-induced expression of SOD. This means that endogenous ANP has participated in the regulation of SOD expression. Results of the current study support previous studies that ANP stimulates antioxidant defense in the cardiovascular system [5] and protects endplate chondrocytes against H_2_O_2_-induced oxidative stress damage by increasing SOD levels and inhibiting apoptotic factors [46].

In summary, CCK-8s promoted ANP secretion through the activation of NOX4–PGC-1*α*–PPAR*α*/PPAR*γ* signaling and played a negative inotropic effect through the activation of K_ATP_ and BK_Ca_. The CCK-8s-induced endogenous ANP was involved in resistance for NOX4 expression and ROS production and regulation of SOD activity. This suggests that the CCK–ANP signaling is implicated in cardiac physiology and pathophysiology.

## Figures and Tables

**Table 1:** Experimental protocols.

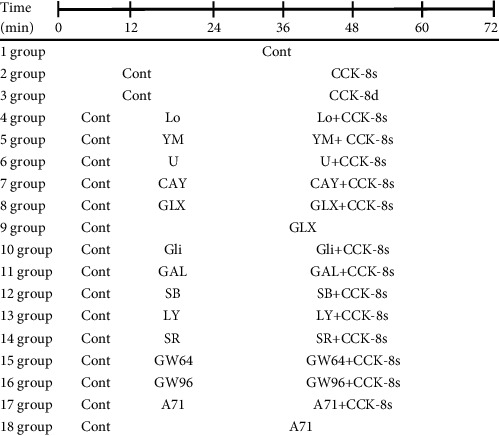

Note: control (Cont); sulfated CCK-8 (CCK-8s); desulfated CCK-8 (CCK-8d); Loxiglumid (Lo); YM022 (YM); U-73122 (U); CAY10650 (CAY); GLX351322 (GLX); Glibenclamide (Gli); GAL-021 (GAL); SB239063 (SB); LY294002 (LY); SR-18292 (SR); GW6471 (GW64); GW9662 (GW96); A71915 (A71).

**Table 2:** Primer sequences.

Gene	Primer sequence
NPPA	S: 5′-TTCCTCTTCCTGGCCTTTTG-3′ A: 5′-TCTAGCAGGTTCTTGAAATCCATC-3′
PPAR*α*	S: 5′-TCCACAAGTGCCTGTCCGTC-3′ A: 5′-CTTCAGGTAGGCTTCGTGGATT-3′
PPAR*γ*	S: 5′-TTTCAAGGGTGCCAGTTTCG-3′ A: 5′-GGAGGCCAGCATGGTGTAGAT-3′
*β*-Actin	S: 5′-TGCTATGTTGCCCTAGACTTCG-3′ A: 5′-GTTGGCATAGAGGTCTTTACGG-3′

Note: S: sense primer; A: antisense primer.

## Data Availability

The data supporting the findings of this study are available from the corresponding author upon reasonable request.

## References

[1] Goetze J. P., Bruneau B. G., Ramos H. R., Ogawa T., de Bold M. K., de Bold A. J. (2020). Cardiac natriuretic peptides. *Nature Reviews Cardiology.*.

[2] Ilatovskaya D. V., Levchenko V., Winsor K. (2022). Effects of elevation of ANP and its deficiency on cardiorenal function. *Insight*.

[3] Cannone V., Burnett J. C. (2022). Natriuretic peptides and blood pressure homeostasis: implications for MANP, a novel guanylyl cyclase a receptor activator for hypertension. *Frontiers in Physiology*.

[4] D'Souza S. P., Davis M., Baxter G. F. (2004). Autocrine and paracrine actions of natriuretic peptides in the heart. *Pharmacology & Therapeutics*.

[5] De Vito P., Incerpi S., Pedersen J. Z., Luly P. (2010). Atrial natriuretic peptide and oxidative stress. *Peptides*.

[6] Laskowski A., Woodman O. L., Cao A. H. (2006). Antioxidant actions contribute to the antihypertrophic effects of atrial natriuretic peptide in neonatal rat cardiomyocytes. *Cardiovascular Research*.

[7] Serafino A., Pierimarchi P. (2014). Atrial natriuretic peptide: a magic bullet for cancer therapy targeting Wnt signaling and cellular pH regulators. *Current Medicinal Chemistry*.

[8] Rehfeld J. F. (2021). Cholecystokinin and the hormone concept. *Endocrine Connections*.

[9] Rehfeld J. F. (2017). Cholecystokinin—from local gut hormone to ubiquitous messenger. *Front Endocrinol (Lausanne).*.

[10] Nässel D. R., Wu S. F. (2022). Cholecystokinin/sulfakinin peptide signaling: conserved roles at the intersection between feeding, mating and aggression. *Cellular and Molecular Life Sciences*.

[11] Saia R. S., Bertozi G., Mestriner F. L., Antunes-Rodrigues J., Queiróz Cunha F., Cárnio E. C. (2013). Cardiovascular and inflammatory response to cholecystokinin during endotoxemic shock. *Shock*.

[12] Han Z., Bi S., Xu Y. (2021). Cholecystokinin expression in the development of myocardial hypertrophy. *Scanning*.

[13] Dockray G. J. (2012). Cholecystokinin. *Current Opinion in Endocrinology, Diabetes, and Obesity*.

[14] Shrivastava S., Jafurulla M., Tiwari S., Chattopadhyay A. (2018). Identification of sphingolipid-binding motif in G protein-coupled receptors. *Advances in Experimental Medicine and Biology*.

[15] Goetze J. P., Johnsen A. H., Kistorp C., Gustafsson F., Johnbeck C. B., Rehfeld J. F. (2015). Cardiomyocyte expression and cell-specific processing of procholecystokinin∗. *The Journal of Biological Chemistry*.

[16] Wang C., Yu H., Wei L. (2020). Protective effect of cholecystokinin octapeptide on angiotensin II-induced apoptosis in H9c2 cardiomyoblast cells. *Journal of Cellular Biochemistry*.

[17] Ruiz-Gayo M., González M. C., Fernández-Alfonso S. (2006). Vasodilatory effects of cholecystokinin: new role for an old peptide?. *Regulatory Peptides*.

[18] Zhao X. Y., Ling Y. L., Li Y. G., Meng A. H., Xing H. Y. (2005). Cholecystokinin octapeptide improves cardiac function by activating cholecystokinin octapeptide receptor in endotoxic shock rats. *World Journal of Gastroenterology*.

[19] Wang C., Zhang C., Wu D. (2020). Cholecystokinin octapeptide reduces myocardial fibrosis and improves cardiac remodeling in post myocardial infarction rats. *The International Journal of Biochemistry & Cell Biology*.

[20] Dong X., Wang C., Zhang J. (2017). Cholecystokinin expression in the development of postinfarction heart failure. *Cellular Physiology and Biochemistry*.

[21] Goetze J. P., Rehfeld J. F., Alehagen U. (2016). Cholecystokinin in plasma predicts cardiovascular mortality in elderly females. *International Journal of Cardiology*.

[22] Zhang Y., Murugesan P., Huang K., Cai H. (2020). NADPH oxidases and oxidase crosstalk in cardiovascular diseases: novel therapeutic targets. *Nature Reviews. Cardiology*.

[23] Wu C. Z., Li X., Hong L. (2021). NOX4/Src regulates ANP secretion through activating ERK1/2 and Akt/GATA4 signaling in beating rat hypoxic atria. *Korean J Physiol Pharmacol.*.

[24] Zeng Q., Ou L., Wang W., Guo D. Y. (2020). Gastrin, cholecystokinin, signaling, and biological activities in cellular processes. *Front Endocrinol (Lausanne).*.

[25] Yoshida H., Tsunoda Y., Owyang C. (1997). Cholecystokinin peptides stimulate pancreatic secretion by multiple signal transduction pathways. *The American Journal of Physiology*.

[26] Pommier B., Da Nascimento S., Dumont S. (1999). The cholecystokininB receptor is coupled to two effector pathways through pertussis toxin-sensitive and -insensitive G proteins. *Journal of Neurochemistry*.

[27] Rozengurt E., Walsh J. H. (2001). Gastrin, CCK, signaling, and cancer. *Annual Review of Physiology*.

[28] Mangum L. C., Borazjani A., Stokes J. V. (2015). Organochlorine insecticides induce NADPH oxidase-dependent reactive oxygen species in human monocytic cells via phospholipase A2/arachidonic acid. *Chemical Research in Toxicology*.

[29] Colston J. T., de la Rosa S. D., Strader J. R., Anderson M. A., Freeman G. L. (2005). H_2_O_2_ activates Nox4 through PLA2-dependent arachidonic acid production in adult cardiac fibroblasts. *FEBS Letters*.

[30] Zhou T., Li S., Yang L., Xiang D. (2021). MicroRNA-363-3p reduces endothelial cell inflammatory responses in coronary heart disease via inactivation of the NOX4-dependent p38 MAPK axis. *Aging (Albany NY)*.

[31] Gao S., Yuan K., Shah A., Kim J. S., Park W. H., Kim S. H. (2011). Suppression of high pacing-induced ANP secretion by antioxidants in isolated rat atria. *Peptides*.

[32] Zhou X., Teng B., Tilley S., Mustafa S. J. (2013). A1 adenosine receptor negatively modulates coronary reactive hyperemia via counteracting A2A-mediated H_2_O_2_ production and K_ATP_ opening in isolated mouse hearts. *American Journal of Physiology. Heart and Circulatory Physiology*.

[33] Hu X. Q., Zhang L. (2012). Function and regulation of large conductance Ca^2+^-activated K^+^ channel in vascular smooth muscle cells. *Drug Discovery Today*.

[34] Zhang D. M., Lin Y. F. (2020). Functional modulation of sarcolemmal KATPchannels by atrial natriuretic peptide-elicited intracellular signaling in adult rabbit ventricular cardiomyocytes. *American Journal of Physiology. Cell Physiology*.

[35] Doukbi E., Soghomonian A., Sengenès C. (2022). Browning epicardial adipose tissue: friend or foe?. *Cell*.

[36] Scharf M., Neef S., Freund R. (2013). Mitogen-activated protein kinase-activated protein kinases 2 and 3 regulate SERCA2a expression and fiber type composition to modulate skeletal muscle and cardiomyocyte function. *Molecular and Cellular Biology*.

[37] Lu Z., Xu X., Hu X. (2010). PGC-1*α* regulates expression of myocardial mitochondrial antioxidants and myocardial oxidative stress after chronic systolic overload. *Antioxidants & Redox Signaling*.

[38] McLeod C. J., Pagel I., Sack M. N. (2005). The mitochondrial biogenesis regulatory program in cardiac adaptation to ischemia--a putative target for therapeutic intervention. *Trends in Cardiovascular Medicine*.

[39] Ibarra-Lara L., Hong E., Soria-Castro E. (2012). Clofibrate PPAR*α* activation reduces oxidative stress and improves ultrastructure and ventricular hemodynamics in no-flow myocardial ischemia. *Journal of Cardiovascular Pharmacology*.

[40] Chen T., Jin X., Crawford B. H. (2012). Cardioprotection from oxidative stress in the newborn heart by activation of PPAR*γ* is mediated by catalase. *Free Radical Biology & Medicine*.

[41] Mezzasoma L., Peirce M. J., Minelli A., Bellezza I. (2017). Natriuretic peptides: the case of prostate cancer. *Molecules*.

[42] Zhang Y., Li X., Liu L. P. (2017). Peroxisome proliferator-activated receptor *γ* is essential for secretion of ANP induced by prostaglandin D2in the beating rat atrium. *The Korean Journal of Physiology & Pharmacology*.

[43] Li X., Zhang Y., Zhang B. (2018). HIF-1*α*-l-PGDS-PPAR*γ* regulates hypoxia-induced ANP secretion in beating rat atria. *Prostaglandins & Other Lipid Mediators*.

[44] Li X., Han Z. N., Liu Y., Hong L., Cui B. R., Cui X. (2019). Endogenous ET-1 promotes ANP secretion through activation of COX2-L-PGDS-PPAR*γ* signaling in hypoxic beating rat atria. *Peptides*.

[45] Dubois-Deruy E., Peugnet V., Turkieh A., Pinet F. (2017). Oxidative stress in cardiovascular diseases. *Antioxidants (Basel)*.

[46] He F., Gai J., Wang J., Tang L., Liu Y., Feng Q. (2021). Atrial natriuretic peptide protects vertebral endplate chondrocytes against H_2_O_2_-induced apoptosis and oxidative stress through activation of the Nrf2/HO-1 signaling pathway. *Molecular Medicine Reports*.

